# The Microfluidic Trainer: Design, Fabrication and Validation of a Tool for Testing and Improving Manual Skills

**DOI:** 10.3390/mi11090872

**Published:** 2020-09-19

**Authors:** Francesco Costa, Luigi Falzetti, Nicola Baldini, Sofia Avnet

**Affiliations:** 1 BST Biomedical Science and Technologies Lab, IRCCS Istituto Ortopedico Rizzoli, 40126 Bologna, Italy; luigi.falzetti@ior.it (L.F.); nicola.baldini@ior.it (N.B.); sofia.avnet@ior.it (S.A.); 2 Department of Biomedical and Neuromotor Sciences, University of Bologna, 40127 Bologna, Italy

**Keywords:** microfluidics, handling skills, in vitro techniques, basic research, education

## Abstract

Microfluidic principles have been widely applied for more than 30 years to solve biological and micro-electromechanical problems. Despite the numerous advantages, microfluidic devices are difficult to manage as their handling comes with several technical challenges. We developed a new portable tool, the microfluidic trainer (MT), that assesses the operator handling skills and that may be used for maintaining or improving the ability to inject fluid in the inlet of microfluidic devices for in vitro cell culture applications. After several tests, we optimized the MT tester cell to reproduce the real technical challenges of a microfluidic device. In addition to an exercise path, we included an overfilling indicator and a correct infilling indicator at the inlet (control path). We manufactured the MT by engraving a 3 mm-high sheet of methacrylate with 60W CO_2_ laser plotter to create multiple capillary paths. We validated the device by enrolling 21 volunteers (median age 33) to fill both the MT and a commercial microfluidic device. The success rate obtained with MT significantly correlated with those of a commercial microfluidic culture plate, and its 30 min-continuous use for three times significantly improved the performance. Overall, our data demonstrate that MT is a valid assessment tool of individual performances in using microfluidic devices and may represent a low-cost solution to training, improve or warm up microfluidic handling skills.

## 1. Introduction

Microfluidics is the science of systems that process and manipulate small quantities of fluids through channels of a few hundred micrometres, in which capillarity phenomena dominate the dynamics of fluids. This feature has led to the development of devices that can control fluid manipulation in a remarkably precise way in many different contexts, like semiconductor industries and in the micro-electromechanical field [1]. However, in addition to physics and engineering, microfluidics has recently embraced other disciplines, like chemistry and biology. Indeed, the same approach has been successfully applied to address complex biology and medicine issues to build in vitro models that are useful to understand cellular and biological processes. Microfluidic devices can simulate the functional units of a tissue or an organ on a small surface (organ-on-a-chip) or can replace and integrate many functions of a lab (lab-on-a-chip). This proved to be particularly advantageous both in basic research and in the diagnostic field. Over the years, many microfluidic devices have been designed and introduced into the market for biomedical applications, like DNA sequencing, functional genomics, single-cell studies [2], cell-to-cell investigation [3], and in several specialized biomedical sectors, like neural tissue engineering [4] and cancer investigation [5], including bone metastasis [6], drug discovery and screening [7], and diagnostics [8].

The main advantages of a microfluidic devices in in vitro disease modelling are: (1) working at a single-cell scale [9,10]; (2) the use of techniques that take into account 3D architecture and chemical/physical microenvironment composition of the referred tissue that cannot be considered in 2D monolayer cell culture [11,12,13,14]; (3) a high level of standardization and reproducibility; (4) a low-budget production [15]; (5) maximization of the information collected starting from small samples, thereby reducing the costs for the experimentation, as compared to conventional benchtop equipment [15]. Miniaturization and cost-cutting for the operator that works in the biomedical field also allow enhancing the scalability and increasing the number of replicates that can be performed in a single assay. Finally, microfluidic devices are an excellent opportunity to reduce the use of animal experiments to overcome the burden of ethical issues. One relevant example is the use of microfluidics for preclinical screening during the drug discovery process [7].

However, despite over the past two decades these devices have been envisioned to be handled on a large scale, their use is not yet widely spread in research institutes and companies. One of the reasons that can be argued is that approaching microfluidic devices might be distressing. The acquisition of novel technologies always requires a very specific training period and new learning curves. Indeed, handling of microfluidic devices comes with several technical hurdles that result from the miniaturization of wells. The procedure of filling in fluids deals with very small-size lines and channels, thereby requiring the handling of very small sample volumes. In particular, the inaccuracies of the quantitative volume handling have been found with a significant increase to more than 20% in the ranges below 1 µL [16,17]. Inevitable operational human errors, inaccuracy in low volume quantitation, incomplete channel filling and, on the opposite, excessive filling, have been standing challenges for the handheld manual micropipetting for microfluidic applications, often resulting in the requirement of additional repeats for the fluid injection in the channel inlets. Smoothness, the precision of movements and eye-hand coordination are crucial for the correct use of microfluidic devices and human errors may pose potential risks of failed experiments, inaccurate results, and financial costs. As such, without an effective means of preliminary training, many experimental results may become unreliable, also due to the extra expense of costly labour, reagents and dispensable microfluidic devices. As a confirmation of the existence of the problem, to address the aforementioned issues there has been a continuous development of the robotic-based liquid handling system to reduce human-induced errors to a large extent [18]. However, also instrumentations for automatic dispensation of fluids may be not always affordable for public and private research and educational laboratories.

As for other manual techniques in the biomedical fields, for the handling of microfluidic devices, brain requires specific training to improve muscle memory and fine movements of the upper limb and hand. In fact, the literature shows examples on how it is possible to train coordination and control of movements, like for manual training for movements needed for microsurgical procedures. In microsurgery, which shares with microfluidics the small size of the structures and the need for micro-coordination, numerous ex vivo training exercises [19,20] have been devised to improve the quality of movement, and training has become a fundamental part of the training of a young microsurgeon [21], improving coordination, tremor compensation, the use of the instruments, and reducing the time of exercising. Similarly, training on microfluidics could represent a helpful tool to improve micro-coordination. However, microfluidic devices cost much more than standard plastic in vitro supports and, to date, there is no gold standard in the practice for microfluidic gesture skill development. As a consequence, many end users might not be willing to change their conventional methods and instruments for time and cost consuming training with uncertainties.

The development of a device at a very affordable price that can be used, both for assessing the level of manual skills of a first-hand operator and for improving the accuracy of gestures, might solve this problem.

In this study, for the first time, we developed and validated a low-cost and simple-to-read device to assess and train the individual handling skills of researchers, technicians and students for the loading of microfluidic channels, especially for those devices that are used for in vitro biological model and that include an inlet to inject fluids, with or without cells.

## 2. Materials and Methods

### 2.1. Design of the Microfluidic Trainer 

**Figure 1:**
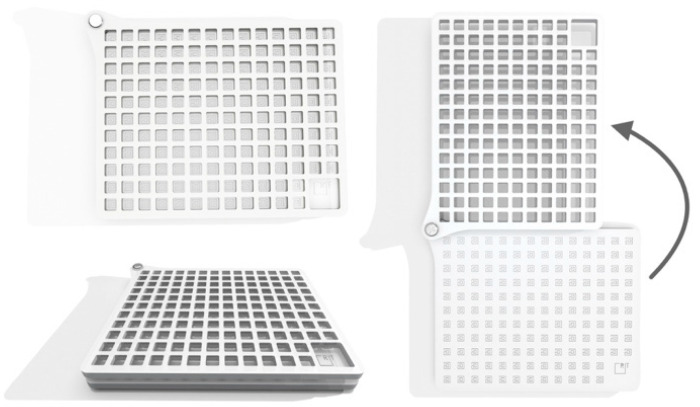
Design of the microfluidic trainer (MT). The closed MT device (left panel). The opened MT device (right panel).

We designed the microfluidic trainer (MT, Figure 1) by using AutoCAD 3D^®^ (Autodesk, San Rafael, CA, USA) and Rhinoceros 6.0 (Robert McNeel & Associates, Seattle, Washington, DC, USA).

We set the dimensions of the device by using a SBS-standard plate/96-weel plate dimensions (127.76 mm × 85.48 mm × 14.8 mm), as a reference.

**Figure 2:**
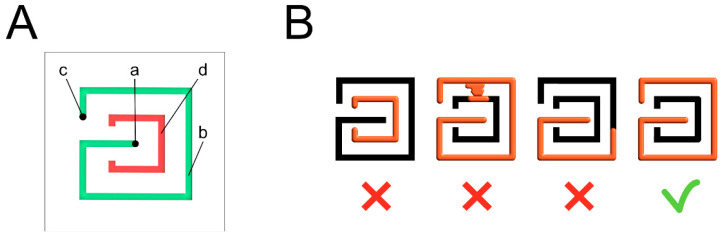
MT single tester cell. (**A**) Inlet (a), exercise path (b), correct filling indicator of the exercise path (c), correct infilling indicator at the inlet (control path) (d); (**B**) Schematic representation of the correct and incorrect loading and filling of the MT single tester cell.

After several testing, we optimized the final design of the tester cell (Figure 2a) and the height of the MT device to pursue two objectives: (1) to reproduce the real technical challenges of a microfluidic device; (2) to maintain a low production cost. The distribution of the tester cells has been developed to have the maximum number in a single exercise plate. The diameter of the inlet and the diameter of the exercise path has been defined to be as similar as possible to those of the reference microfluidic plate that has been considered in this study (OrganoPlate^®^ 2-lane, Mimetas, Leiden, The Netherlands). At the inlet, the control path assesses the precise infilling and the loss of fluids at the loading of the channel. 

The overfilling indicator assesses the overloading of the channel (exercise path). For a scheme of the correct and incorrect fillings of the MT single tester, see Figure 2b.

### 2.2. The Manufacturing the Microfluidic Trainer 

**Figure 3:**
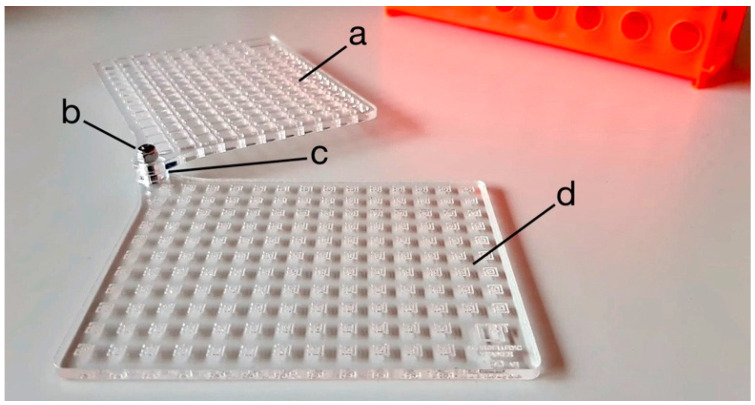
Components of the MT device. Cover grid (**a**), self-locking screw (**b**), spacer ring (**c**), exercise plate (**d**).

We manufactured the exercise plate and the cover grid by laser cutting a 3 mm high methacrylate sheet. We then assembled the exercise plate, a spacer ring, and the cover grid by using a self-locking screw (Figure 3). We manufactured the MT tester cells by engraving and slicing a methacrylate sheet (60W CO_2_ laser plotter, LB7050, Laser Bros, Florence, Italy). When laser plotters engrave curved lines, small irregularities can form at the border of the channels. These irregularities should be avoided to not interfere with fluid flow and with capillarity. Thus, the exercise path in the exercise plate has been made of multiple rectilinear segments to simplify the manufacturing by laser plotter.

We included a spacer ring in the device (diameter and thickness 1.5 mm and 3 mm, respectively) to keep the lower part distant from the upper part, thereby avoiding the deburring of fluids by capillarity that can occur during the procedures of loading the MT tester cells with fluids, from the testing plate to the cover grid. The spacer ring and the cover grid were added also to increase the technical challenge. The aim was to simulate the spacers in the microfluidic plates that do not allow to keep the micropipette in the optimal position to load the inlet.

We set up the parameters of the laser plotter to cut off the borders of the cover grid and the exercise path, and to form the holes in the cover grid, as follows: speed 6.0 and power 68%. We cut the cover grid to obtain 150 holes. We then engraved the upper surface of the exercise plate to obtain 150 tester cells by adjusting the parameters of the laser plotter, as follows: speed 30.0% and power 18%. To form a single tester cell, we engraved two separate paths: a G-like exercise path made of 6 segments (b in Figure 2a) and a C-like control path made of 5 segments (d in Figure 2a). The cutting process took around 150 s for each device. In the final prototype, the resulting channels had a 0.4 mm width and a 0.2 mm depth. This miniaturized volume allows the fluids to flow in the channels by capillarity.

Finally, during the assembling, we aligned the cover grid and the exercise plate to face each single tester cell on the exercise plate to the corresponding hole in the cover grid. For the assembling, we fixed a self-locking screw in the upper left corner of the MT device.

### 2.3. The Pilot Study to Validate the Microfluidic Trainer as a Tester (Pilot Study 1)

We validated the MT device as a tester by estimating how much the handling technical difficulties are similar to those of a commercial microfluidic plate for in vitro cell culturing (OrganoPlate^®^ 2-lane, Mimetas). 

After informed consent, we randomly enrolled 21 volunteers (female 15, male 6, median age 33). Exclusion criteria: previous experience in using microfluidic devices. All subjects were requested to fill both the MT device and a commercial microfluidic device by following a prefixed validation protocol. For the study protocol, 11 volunteers used the MT device before the commercial microfluidic device, and 10 volunteers did the opposite. All subjects were equally hinted with some practical advice on how to handle the micropipette (i.e., inclination, thumb sensibility) and on how to fill in the channels. We showed the self-explanatory scheme (Figure 2b) for the MT device or the manufacturer instruction for the commercial microfluidic plate. To reduce the hand tremors, the leaning of the tip against the margins of the inlet or the holding the tip with the non-dominant hand were allowed.

Finally, to collect additional data, the volunteers were asked to complete a questionnaire on the type of degree and on the number of months of previous experience in bench work or in micropipetting during the past 6 months. As an index of the level of experience in handling a microfluidic device, we considered the percentage of correctly filled cells and channels in respect to the total practised cells and channels, in the MT device and the commercial microfluidic plate, respectively.

**Figure 4:**
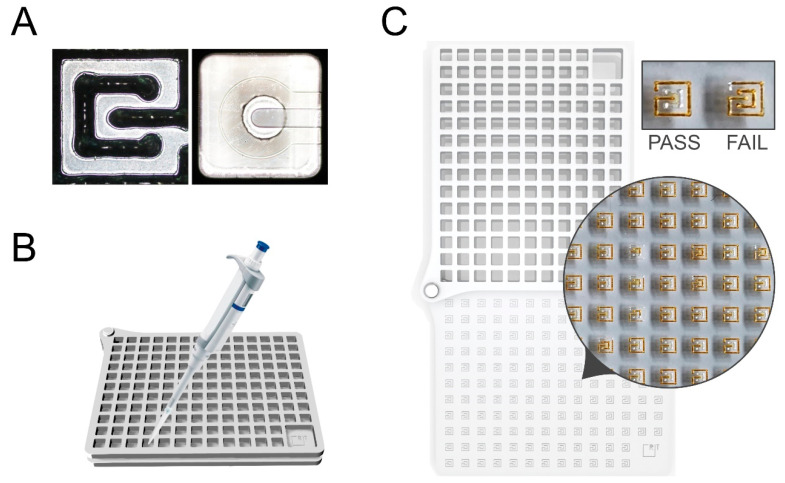
Validation protocol. (**A**) Comparison of the inlet of the MT single tester cell (on the left) and of the commercial microfluidic plate (OrganoPlate^®^ 2-lane, on the right) (objective 4×); (**B**) Graph representation of the filling process of the MT device; (**C**) MT tester cells on the exercise plate that are filled with the staining solution (1.3 µL Betadine^®^), at the end of the validation test (representative image). On the top right, a magnification of representative images of one correctly filled cell (pass), and one failed cell (fail).

To validate the MT device, we then analysed the correlation between the two values. In Figure 4A, we showed the comparison of the inlet in the MT plate and the selected commercial microfluidic plate, under the optical microscope.

All the procedures were performed under a laminar flow cabinet and by using a 10 µL Eppendorf Research^®^ plus single-channel pipette (Eppendorf, Hamburg, Germany). The MT device was always used in the ‘closed’ position. 1.5 µL of a stained solution with at the suitable viscosity (Betadine^®^, Mylan, Canonsburg, PA, USA) were dispensed in the inlet of 30 MT tester cells, or 1.3 µL of sterile distilled water were dispensed in the inlet of 30 cells in the commercial microfluidic device. 

The percentage of correctly filled tester units were counted by eyes for the MT testers or by using an inverted optical microscope (Nikon, Tokyo, Japan) for the commercial microfluidic device, respectively. Correctly or incorrectly filled tester cells were distinguished in the MT device according to the scheme of Figure 3, or as showed in Figure 4C, whereas for the commercial microfluidic plate we followed the manufacturer instructions.

### 2.4. The Pilot Study to Validate the Microfluidic Trainer as a Trainer (Pilot Study 2)

We then validated the MT device as a trainer through a second pilot study, to estimate how much the exercise of repeating the correct infill of the MT cells improves the ability of using a commercial microfluidic plate for in vitro cell culturing (OrganoPlate^®^ 2-lane, Mimetas). 

After informed consent, we randomly enrolled 8 volunteers (female 7, male 1): 3 experts that had already used a microfluidic device for in vitro biological modelling in the past six months, and 5 new users. To assess the success rate at T0, all subjects were requested to fill 30 cells of the OrganoPlate^®^ 2-lane (Mimetas) at the beginning of the protocol. We showed the manufacturer instruction for the commercial microfluidic plate. Of the 30 cells, the operators had to inject: for the new user, 1.2 µL of distilled waters for 20 cells and 2 µL of collagen (Cultrex 3-D Culture Matrix Rat Collagen I, R&D Systems, Minneapolis, MN, USA) for 10 cells; for the expert, 1.2 microliters with distilled waters for 10 cells and 2 microliters of collagen for 20 cells. Collagen was used in place of water to increase the degree of challenge of the testing, especially for the experts. All subjects were equally hinted with some practical advice on how to handle the micropipette (i.e., inclination, thumb sensibility) and on how to fill in the channels. As an index of the level of experience in handling a microfluidic device, we considered the percentage of correctly filled cells and channels with respect to the total practiced cells and channels. The percentage of correctly filled tester units in the MT device were counted by eye (Nikon) (Figure 4C). Correctly or incorrectly filled tester cells in the commercial microfluidic plate were distinguished according to the manufacturer instructions.

After the initial testing, the operators performed three slots of continuous training, 30-min long each, by using the MT device and according to the procedure described in Section 2.3. The three slots were equally distributed over a day-and-a-half period of time. To reduce the hand tremors, the leaning of the tip against the margins of the inlet or the holding the tip with the non-dominant hand were allowed. Soon after the third slot of training (T1), the volunteers were asked to repeat the protocol of asses the success rate with the commercial microfluidic device, as it was performed at T0. To validate the MT device as a trainer, we then performed a paired analysis of the success rates at T0 and at T1. 

### 2.5. Statistical Analysis

Statistical analyses were performed using GraphPad Prism version 7.04 for Windows (GraphPad Software, La Jolla, CA, USA). Due to the low number of observations, we considered data as not normally distributed and we thus used nonparametric tests. We used the Spearman Rank (one tail) for the analysis of the correlation between two continuous variables, and the Wilcoxon test for the correlation between a qualitative variable and a continuous variable (one tail). To evaluate the predictive performance of the MT device, in the receiver operating characteristic (ROC) curve, we used the 65th percentile of the success rate of the selected commercial microfluidic plate as a threshold value to distinguish bed or good performers. Only *p <* 0.05 was considered as statistically significant.

## 3. Results

After completing the validation protocol, in addition to measuring the indexed performances obtained with the MT device and with the commercial microfluidic plate, for each volunteer enrolled in the Pilot study 1, we collected and analysed all information regarding the type of degree, the previous experience that could have improved the manual skills in handling microfluidic supports, and that could be correlated to the level of experience, as measured by the MT device (Table 1). 

**Figure 5:**
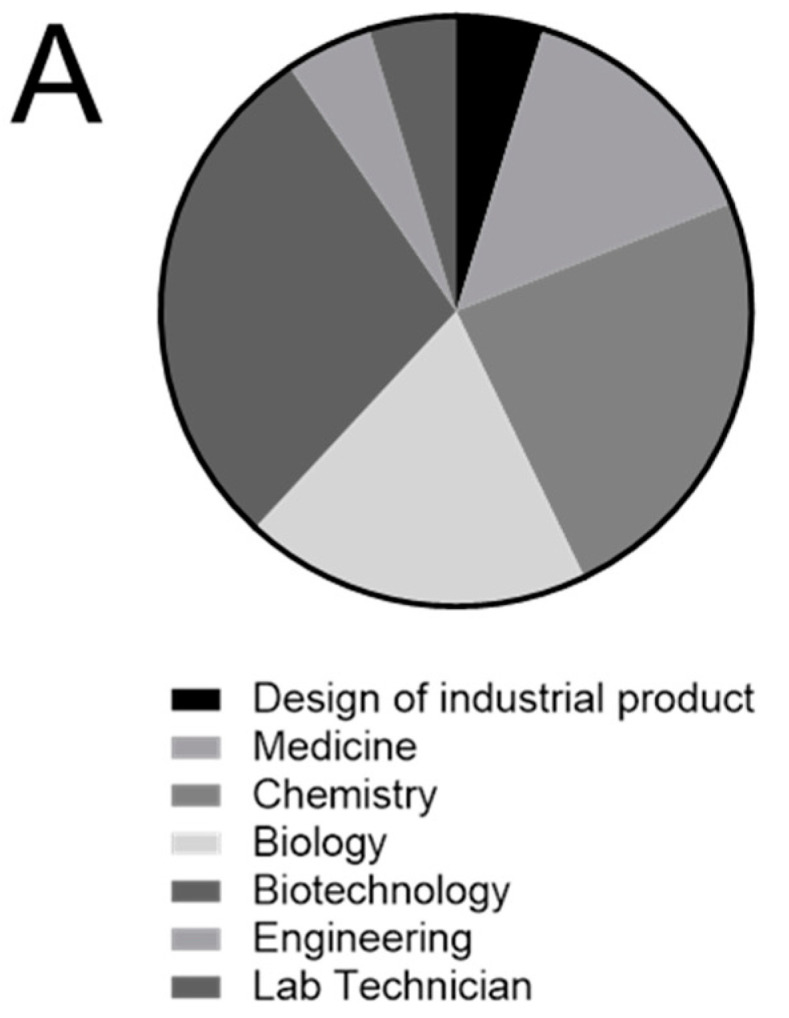

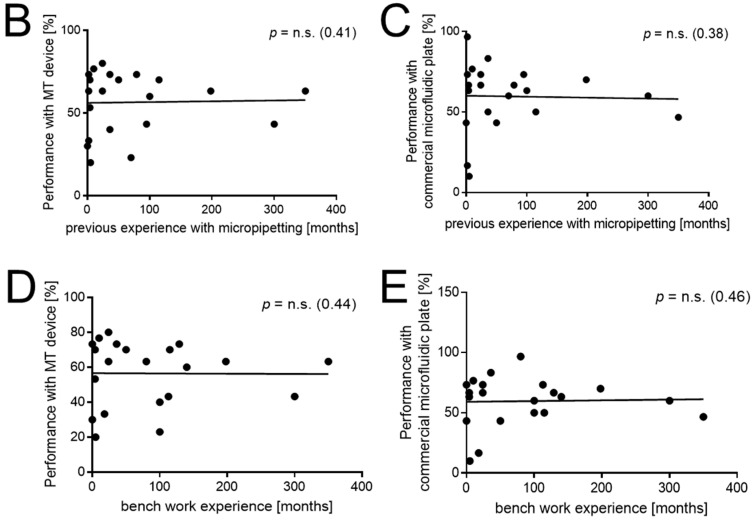
Cultural background and previous experience in micropipetting of volunteers enrolled in Pilot study 1. (**A**) Type of degrees of the volunteers enrolled in Pilot study 1 (*n* = 21); (**B**) Correlation between previous experience in micropipetting and performance with MT device (%); (**C**) Correlation between previous experience in micropipetting and performance with commercial microfluidic plate [%]; (**D**) Correlation between previous experience in bench work and performance with MT device [%]; (**E**) Correlation between previous experience in bench work and performance with commercial microfluidic plate [%]. n.s., not significant.

The types of scientific degree were quite uniformly distributed in the group of volunteers (Figure 5A), and it was thus not considered as a confounding variable.

**Figure 6:**
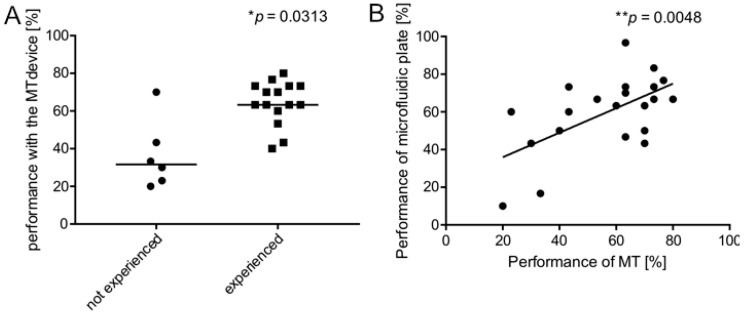

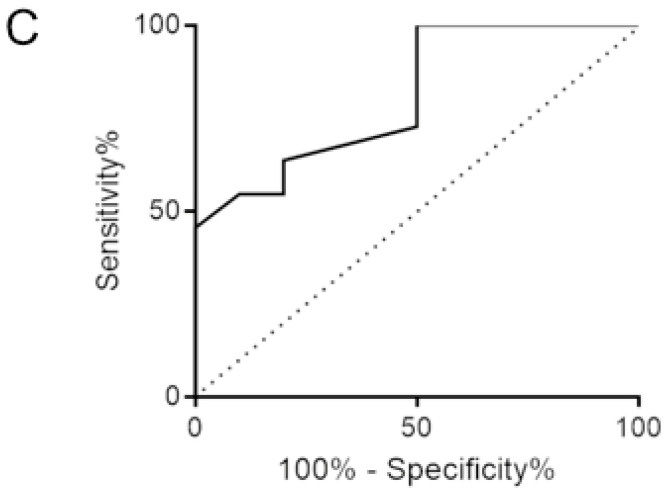
Pilot study 1. Performance of the MT device as a tester. (**A**) Correlation of success rate relative to the MT device with previous recent experience with micropipetting in the last 6 months (*n* = 21, * *p* > 0.05, Wilcoxon correlation test); (**B**) Correlation of success rate relative to the MT device with the level of experience in handling microfluidic supports, as measured with OrganoPlate^®^ 2-lane (*n* = 21, ** *p* < 0.05, Spearman correlation test). (**C**) The receiver operating characteristic (ROC) curve for the prediction ability of the MT device about the performance in handling microfluidic supports (*n* = 21, * *p* = 0.0167, good performers *n* = 10, bed performers *n* = 11).

Age of volunteers did not correlate with the performance on the two plates. Furthermore, neither the score obtained with the MT device nor with the commercial microfluidic device did not correlate with the previous experience in micropipette handling (Figure 5B,C), or with previous experience in bench-work (Figure 5D,E). Adversely of note, the recent usage of micropipettes (during the 6 months before the enrolment) significantly correlated with the performance with the MT device (Figure 6A).

Most importantly, the level of experience in handling microfluidic supports that was obtained with the MT device significantly correlated with that obtained with the selected commercial microfluidic plate (Figure 6B).

Finally, in the attempt to quantify the prediction ability of the MT device to recognize bad or good performers in handling microfluidic supports, we found a cut-off value (66.67%) based on the distribution of values collected with the commercial microfluidic plate in the tested group of volunteers. By using the ROC curve analysis, we obtained sensitivity and specificity of the MT index applied to the commercial microfluidic device that corresponded to an area under the curve of 0.8091 (95% confidence interval: 0.621–0.9941, *p* = 0.0167, z value Hanley and McNeil method = 3.136842, Figure 6C). These data suggest the MT device has a good prediction performance.

**Figure 7:**
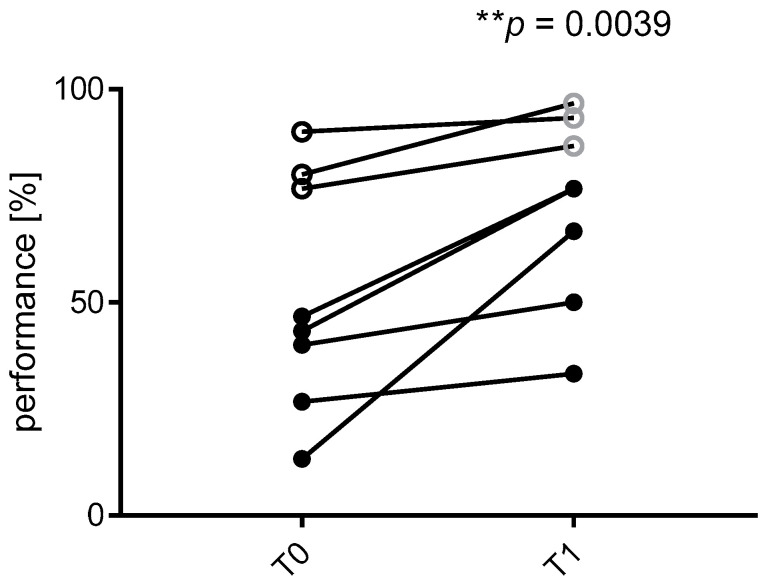
Pilot study 2. Performance of the MT device as a trainer. Level of experience in handling microfluidic supports, as measured with OrganoPlate^®^ 2-lane, before (T0) and after (T1) 3 slots of 30-min continuous training with the MT device. The filled circles are relative to new users, the empty circles are relative to the experts (*n* = 8, ** *p* < 0.005, Wilcoxon test of paired analysis).

By performing a second Pilot study (Pilot study 2) and by enrolling additional 8 volunteers, we also assessed the training potential of the MT device. The volunteers were asked to perform 30 min of continuous exercise with the MT device for three times within a day and a half, and their success rate of correctly filling the microfluidic channel in a commercial microfluidic device was compared before and soon after the training activity. Notably, although we cannot exclude that the second time use of the commercial microfluidic device might have had an impact on the handling improvement, it seemed that the very short training period with the MT device was sufficient to significantly improve the handling skills, both for the new users and for the experts (Figure 7).

## 4. Discussion

Microfluidic supports are generally a set of micro-channels etched or into a material (glass, silicon or polymer) that allows the manipulation of fluids at the submillimeter scale. The first devices were developed by the semiconductor industry and later incorporated into micro-electromechanical systems (MEMS). Recently, the same technology has been applied to plenty of different fields, including biological research for the development of physiologically relevant in vitro models [22,23].

Similarly, in our lab, we recently approached microfluidic devices for the in vitro modelling of bone physiology and disease [13]. In contrast to the advantages of using such devices, there are some technical difficulties in the practical application of this technology. This is also the reason why digital microfluidics [24], or automatic pipetting [18], or automatic dispenser to handle and inject small volume [25,26], have been suggested, also with the aim to minimizing loss of sample. Indeed, for a beginner, the filling of the microfluidic channel can be extremely challenging. Notable, to successfully load fluid into the device and to avoid the formation of bubbles or the overload of the channels, eye-hand coordination and precise control of thumb muscles are needed. More precisely, this difficulty relates primarily to the precision that is required in the movement of the thumb, which must exert an extremely controlled pressure, and secondly to the precision of the positioning of the tip and the control of the tremor. Today, the market offers various devices to partially or completely automate the filling of microfluidic devices, ranging from automated pipette discharge systems (e.g., electronic pipette) to automated loading systems (e.g., pipetting robots) [27,28]. However, these solutions are expensive and not always accessible to all the laboratories. Furthermore, although the automation is a valid productivity ally, it should not be considered as a shortcut to the acquisition of manual skills, especially during the training.

We thus imagined and developed a tool, the MT device, to evaluate operators’ ability, i.e., to define if the operator is ready to use commercial microfluidic devices or if he/she needs more training. In case of need, the device should also allow the training of the same operator. We intended to partially mimic handling microsurgery trainers that have been quite recently developed to improve the efficacy of micro-movements, also through the use of self-assessing tools [29]. However, we wanted to avoid the need of digital simulators or costly instrumentations and devices. We intended to reproduce similar handling sensations of standard microfluidic devices, as well as to gain practical advantages that could make it ideal both for assessing and training purposes, including the possibility to be safely used outside the lab, and by which it is possible to evaluate the success rate for the self-learning process with no need of laboratory instruments. We set up the dimension of the inlet, as well as of the entire device (height and width), in a way that resembles those of the standard 96-well dish. We designed a cover grid to mimic the technical difficulties at the inlet site of many commercial devices. Indeed, when the cover grid is used in the closed position, the fluid must be loaded into walls of methacrylate that is a few millimetres high, and the process of loading is more challenging since both the allowed tilt angle of the micropipette and visibility are reduced. As already specified, we designed the tester cells with various useful feed-back elements that enhance the process of self-learning and auto-correction. For instance, in addition to the exercise path, we inserted a control path and an overfilling indicator to allow the evaluation of the correct infilling at the inlet level and of the entire exercise path.

We prototyped this device using a laser plotter on methacrylate plates. We chose this printing technology due to its fine resolution, high production speed and low costs which, for this type of device, makes it more suitable than the additive 3D printing or the DLP printing. We also selected methacrylate as a suitable material rather than polycarbonate or PVC to reduce the emission of toxic fumes during the cutting process.

After the design and the manufacturing, we verified that the dimensions and capillarity properties of the MT lanes are comparable to those of commercial microfluidic devices. The manufactured MT was also easily washable, a considerable economic advantage for repeated use.

We then set up a first pilot study to verify the potential of the MT device to predict the success rate of loading wells of a microfluidic plates. We used, as a reference, a commercial microfluidic cell culture plate that is widely used in different applications, like the mimicking of the glomerular filtration barrier or of the neuron networks to study cell-derived dopaminergic neurons, or to measure vascular permeability or the integrity of the intestinal epithelium [14,15,16,17,18].

We enrolled inexperienced volunteers in microfluidics, and with different degrees of previous experience in the use of micropipettes. We used Betadine^®^ to rapidly evaluate the filling of the lanes, directly by sight, without the use of the microscope. Indeed, this solution has the optimum low surface tension for the infilling of micrometric channels and intense staining propriety and can be safely handled. As a result, we demonstrated that the levels of manual skill, as quantified by the success rate in filling the MT (MT index) significantly correlated with the previous short-term (6 months) experience in micropipetting rather than with a long-term previous experience. Adversely, we did not find any correlation between the MT index and the type of degree achieved or age. Most importantly, the MT index proved to be predictive in defining the levels of manual skill in filling a commercial microfluidic plate.

Then, we set up a second pilot study to assess if the MT device can be considered also for training purposes. Indeed, when using in vitro microfluidic biological models for new/experienced users, developing/maintaining handling ability is crucial. Indeed, also for experts, standby periods regarding the use of microfluidic devices may occur (i.g., vacation, parental leave, temporary switching to another type of experimental approach) and may be detrimental. At the same time, excessive discarding of valuable biomaterials or expensive consumable during microfluidic training should be avoided. Previous literature on the use of manual skill trainers for fine motor abilities for surgery applications reported that, in a single-blinded randomized study, just 10 min of warm up using an a training box were sufficient to improve dexterity of surgical residents in laparoscopic skills [30]. In this study, we experimented 90 min of training (three continuous slots of 30-min that were spread over a day and a half). As a result of our study, we demonstrated that the MT device can be successfully used to improve the performance of the microfluidic user, at all stages of experience, and in a very short period of time.

## 5. Conclusions

We designed the MT with the intent to find the right spot between the most precise reproduction of the technical difficulties of microfluidic devices and the low production costs.

Through validation studies, we demonstrated that the developed MT can predict the performance of a new user in the liquid filling into the inlet of commercial microfluidic devices to be used for in vitro biological assays, and can improve the performance of both new and expert users. MT is thus a novel, easy-handling, and safe device that is suitable for assessing the manual skills, practising and warming up with microfluidic devices of a large numbers of new users, thereby avoiding a significantly waste of economic resources.

## Figures and Tables

**Table 1:** Level of experience in handling microfluidic supports of volunteers enrolled in Pilot study 1.

Age	Sex	Bench Work Experience (Months)	Previous Experience of Micropipetting (Months)	Pipetting in the 6 Previous Months	MT (%)	Commercial Microfluidic Device (%)
47	F	100	70	No	23.33	60.00
35	F	113	95	Yes	43.33	73.33
32	F	100	36	Yes	40.00	50.00
36	M	18	2	No	33.33	16.67
30	M	0	2	Yes	73.33	73.33
41	M	129	79	Yes	73.33	66.67
33	F	140	100	Yes	60.00	63.33
28	M	4	4	Yes	70.00	63.33
33	F	80	2	Yes	63.33	96.67
25	F	0	0	No	30.00	43.33
61	F	300	300	No	43.33	60.00
41	F	198	198	Yes	63.33	70.00
62	F	350	350	Yes	63.33	46.67
27	F	36	36	Yes	73.33	83.33
22	M	10	10	Yes	76.67	76.67
26	M	4	4	Yes	53.33	66.67
25	F	50	50	Yes	70.00	43.33
25	F	5	5	Yes	20.00	10.00
39	F	115	115	No	70.00	50.00
30	F	24	24	Yes	80.00	66.67
28	F	24	24	Yes	63.33	73.33
